# Eco-friendly transparent poplar-based composites that are stable and flexible at high temperature

**DOI:** 10.1039/c9ra03550h

**Published:** 2019-07-11

**Authors:** Weihua Zou, Delin Sun, Zhangheng Wang, Ruoyao Li, Wenxuan Yu, Pingfang Zhang

**Affiliations:** Central South University of Forestry and Technology Shaoshan South Road 498 Changsha 410004 China sundelin1966@163.com weibick@sina.cn

## Abstract

Farmed poplar could meet the human demand for transparent wood-based composites to replace glass, avoiding the consumption of natural forest resources. We removed the lignin of poplar using a potassium hydroxide (KOH) and deionized water solution, the waste black liquor could be converted into compound potassium fertilizer after being neutralized by phosphoric acid. Polyurethane (PU) was then added to the lignin-stripped poplar and hardened, the transparent poplar-based composite (TPC) has stable transparency at high temperatures, and flexibility – it elongates (about 15%) before breaking. These properties could provide more uses in hot environments requiring a flexible shape. The TPC PU provides transmittance of 85%, haze of 83%, and anisotropic light diffraction.

## Abstract 2

Farmed poplar could meet the human demand for transparent wood-based composites to replace glass, avoiding the consumption of natural forest resources.

## Graphical abstract

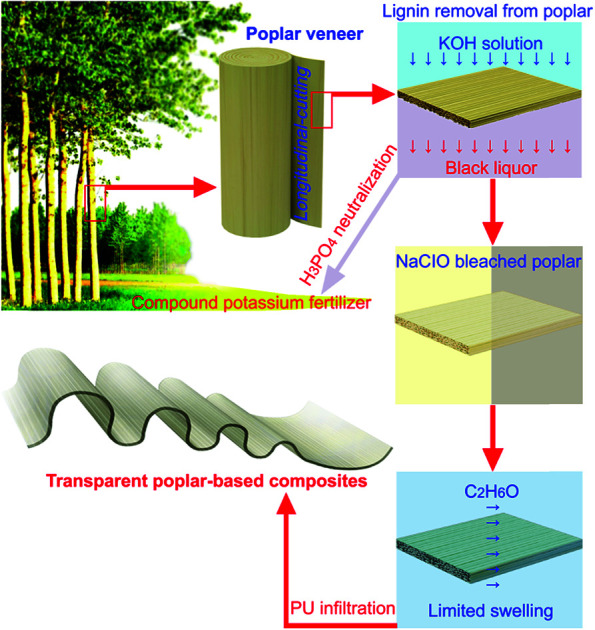

## 1. Introduction

To compete with glass, transparent wood-based composites (TWC) have been introduced as eco-friendly building materials, owing to their high optical transmittance and low thermal conductivity. When replacing glass as a building material, TWC can effectively save energy by guiding natural light into a building, reducing the use of artificial light. TWC can also significantly reduce greenhouse gas emission by insulating and reducing the use of air conditioning.^1–3^ TWC is a kind of cellulose-based biocomposite, whose favourable properties and renewability are important drivers for applications ranging from smart furniture to solar cells.^4,5^ However, if TWC is obtained using forest resources, its eco-friendly material properties will be diminished. Therefore, the raw material selection of TWC must conform to the principle of rational use of natural resources, in order to ensure its original intention to aid eco-friendly and sustainable development.

Poplar is the most widely distributed and adaptable tree species in the world, and it is mainly distributed in the temperate and cold temperate regions of the Northern Hemisphere, latitude 22–70° N, from low altitude to 4800 m. Poplar is mainly distributed in Russia, China, Canada, the United States, Italy, France and so on.^6–8^ Poplar is an important agro-forestry tree in many nations due to its fast growth rate, short rotation period, multiple uses and high economical value.^9^ Responsible use of farmed poplar can not only meet the human demand for TWC, but also avoid the consumption of natural forest resources. Therefore, poplar is used as the raw material of TWC, so that the transparent poplar-based composites (TPC) are always in line with the original intention of environmental protection and sustainable development. The lignin content of poplar is about 20–24% at a low level, and the physical and mechanical characteristics of poplar can be improved by adding polymers.^10^

**Fig. 1:**
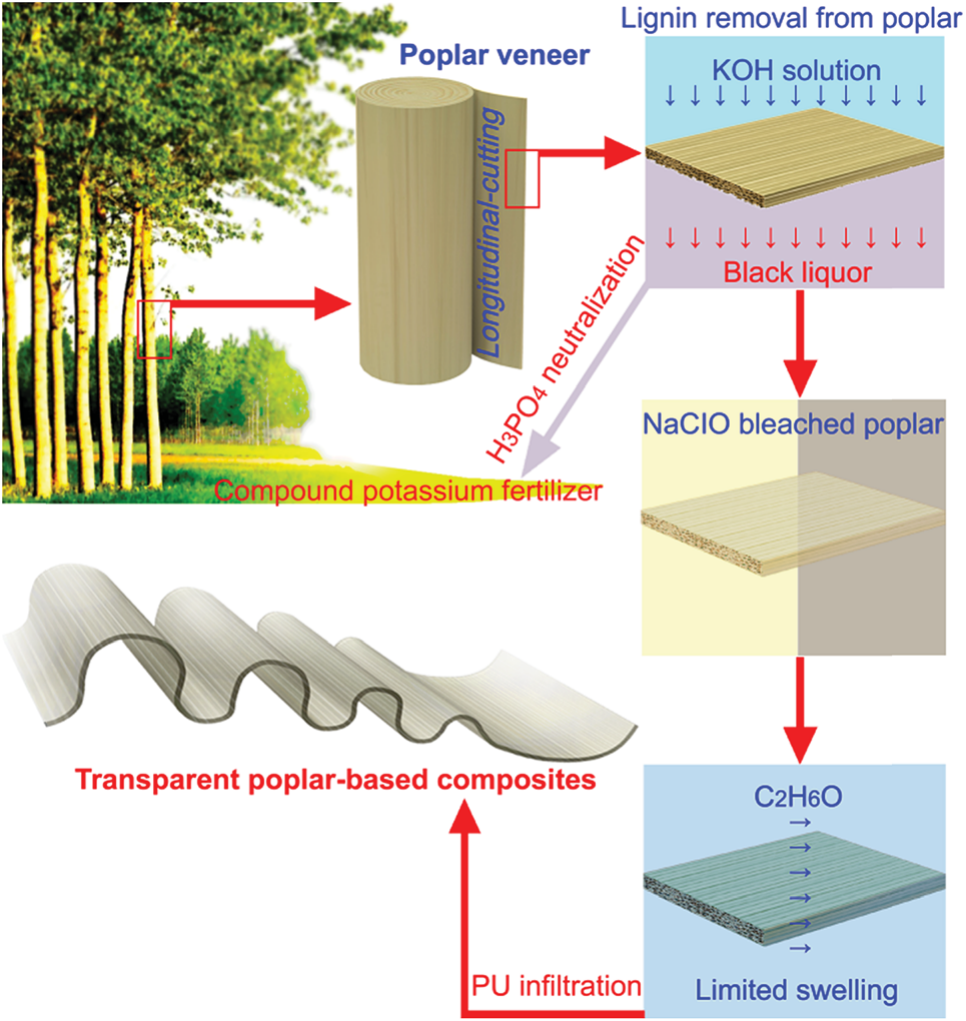
Longitudinal-cutting poplar veneer (PV) was selected and used from fast-growing farmed poplar trunk. KOH and deionized water were used to remove the lignin from the poplar, the resulting black liquor could be recycled into fertilizer. Sodium hypochlorite (NaClO) solution was used to bleach the sample of lignin-stripped PV, and ethyl alcohol absolute (C_2_H_6_O) was used to improve the limited swelling of its cellulose fiber network. Final addition of PU and hardening provides TPC PU, which can be used for applications requiring hot environments and a flexible shape.

The production of TWC comprises the following steps: removing the lignin, bleaching its cellulose fiber network, and adding a transparent polymer that infiltrates the cellulose fiber network.^11–13,15^ The production of TPC mainly follows the above steps (Fig. 1). However, potassium hydroxide (KOH) is used to replace sodium hydroxide (NaOH) for lignin removal from poplar, the black waste liquor could be converted into compound potassium fertilizer after being neutralized by phosphoric acid (H_3_PO_4_),^14^ which reduces the environmental pollution caused by the production process. Polyurethane (PU) is used to replace epoxy resin as the infiltrating polymer to make TPC PU. Compared with TWC, TPC PU has improved flexibility and stability at high temperature. The transparency only had subtle changes after it was treated by thermostatic hot air drying at 90 °C for 100 h, and it has better elongation (about 15%) before breaking. TPC PE provides transmittance of 85%, haze of 83%, and anisotropic light diffraction.

## 2. Experimental

### 2.1 Materials and chemicals

Longitudinal-cutting poplar veneer (PV, 80 mm × 80 mm × 3 mm) was selected and used in this study. As Table 1 shows, the sample of PV had lignin removed in a solution that included potassium hydroxide (KOH, >98%) and deionized water. Sodium hypochlorite (NaClO, >98%) and deionized water were then used to bleach the lignin-stripped PV. Ethyl alcohol absolute (C_2_H_6_O, >99.5%) was used to improve the limited swelling of the cellulose fiber network. PU was then added as the infiltrating polymer. KOH, NaClO, deionized water and C_2_H_6_O were purchased from Aladdin Biochemical Technology (Shanghai, China). PU and its hardener (polyisocyanate) were purchased from Wuhui Port Adhesive Co., Ltd. (Hangzhou, China).

**Table 1:** The chemical formula and method for preparing TPC PU

Method	Chemicals (g, ml)	Temperature (°C)	Time (h)
Lignin removal	KOH (75.74 g), deionized water (500 ml)	120–130	8
Bleach of PV	NaClO (6 g), deionized water (100 ml)	15–25	24
Swelling of PV	C_2_H_6_O (100 ml)	15–25	24
PU infiltration	PU (50 ml), its hardener (50 ml)	25–30	24

### 2.2 Delignification

The sample of PV was immersed in solution that included KOH (2.7 mol L^−1^ in deionized water). After the solution had been boiled for 8 h at 120–130 °C, the sample of PV was removed and the chemicals rinsed off in hot distilled water. After removing a large proportion of lignin, the sample of PV was immersed in NaClO solution (0.81 mol L^−1^ in deionized water) for about 24 h at 15–25 °C until its color disappeared. Then, the PV was rinsed in hot distilled water again.

### 2.3 Limited swelling of cellulose fiber network

Although its cellulose fiber network had been partially swollen during the removal of its lignin and bleaching its cellulose fiber network, the sample of PV was been immersed in C_2_H_6_O (>99.5%, 100 ml) for 24 h at 15–25 °C in order to maintain the existing swelling effect and retain the cellulose fiber network with limited swelling.

### 2.4 Polyurethane infiltration

After PU had been heated at 45–50 °C for 5 min, PU and its hardener were mixed at a ratio of 1 to 1 (PU 50 ml, its hardener 50 ml), and this liquid resin (100 ml) immersed the sample of delignified PV. The liquid resin filled the cellulose-swollen structure of PV and its lumen in an RV-620-2 vacuum reactor (YBIF, Shanghai, China) at 25–30 °C. All the above processes should be completed within 30 min. This kind of poplar–polymer composite was solidified at 25–30 °C for 24 h, and its weight reached ∼20.5 g from ∼8.5 g before PU infiltration.

## 3. Results and discussion

### 3.1 Improving eco-friendliness by utilising waste material

Poplar is a kind of eco-friendly raw material of TWC. Its fast growth rate, short rotation period, multiple uses and high economical value allow it to meet human demand, and can avoid the consumption of natural forests. Previous research focuses on TWC from radial-cutting veneer for its easier in delignification, however, in our work, the TPC from longitudinal-cutting PV could obtain far larger breadth from the poplar trunk and better mechanical properties.

**Fig. 2:**
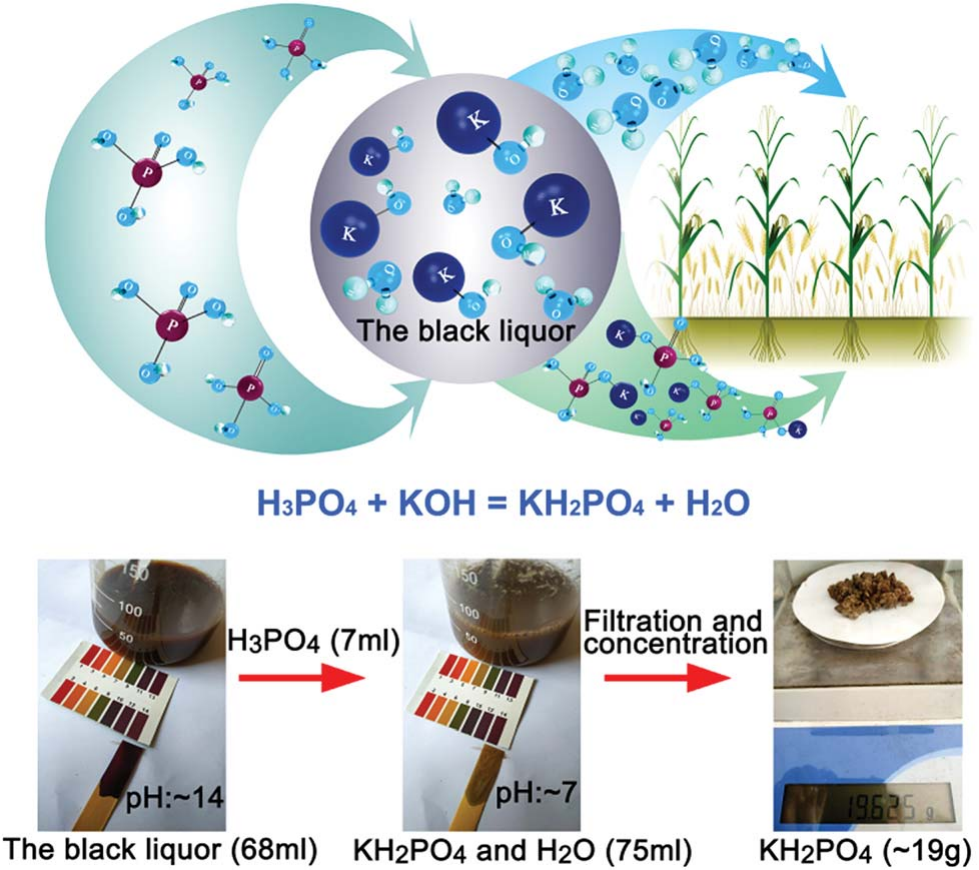
The black liquor could be converted into a compound potassium fertilizer (KH_2_PO_4_) and water (H_2_O).

KOH is a kind of eco-friendly delignified material used for lignin removal from poplar. As shown in Fig. 2, the black liquor of KOH can be neutralized by H_3_PO_4_, and a compound potassium fertilizer (KH_2_PO_4_) can be prepared by filtering and concentrating with a five-effect evaporator.^14^ In our work, the black liquor of KOH (68 ml) whose pH was adjusted from ∼14 to ∼7 by adding H_3_PO_4_ (7 ml, >98%), then, KH_2_PO_4_ (∼19 g) was prepared by filtration and concentration.

### 3.2 Modification of cell wall contents and microstructure

During delignification and bleaching, the sample of PV has reduced water-absorbent properties and the hydrophobicity is obviously enhanced. Therefore, C_2_H_6_O was used to improve the limited swelling of the cellulose fiber network more than water. C_2_H_6_O can also dissolve residues other than cellulose.

**Fig. 3:**
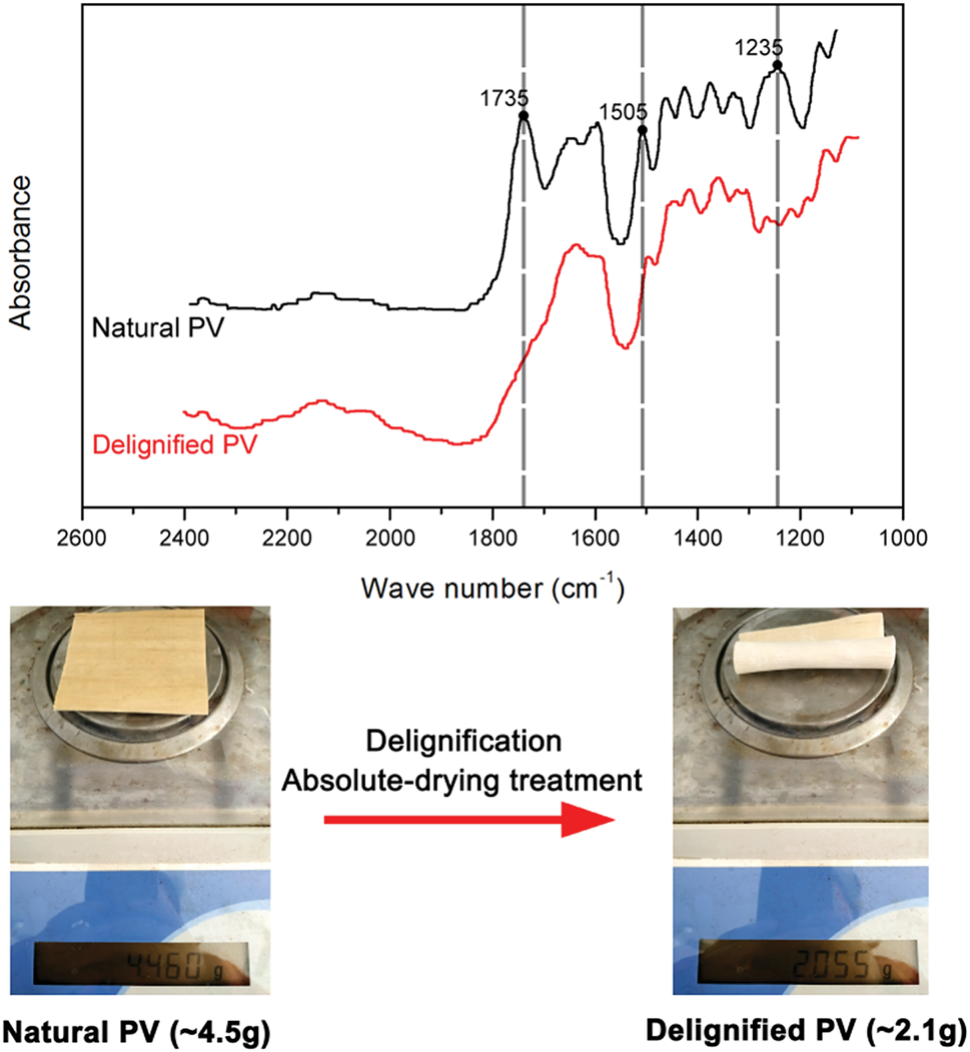
FTIR spectra and the absolute-drying weight from natural PV to delignified PV.

Fourier transform infrared spectroscopy (FTIR) was used to investigate the changes of lignin from natural PV to delignified PV. FTIR spectra were performed by FTIR-850 (Gangdong, Tianjin, China). In the FTIR spectrum, the band at 1505 cm^−1^ is characteristic of aromatic compounds (phenolic hydroxy groups) and is attributed to aromatic skeleton vibrations from lignin.^16,17^ The band at 1235 cm^−1^ can be associated with hemicelluloses, and the band at 1735 cm^−1^ represents C=O functional group.^18–20^ To compare with natural PV, the peaks of delignified PV at 1505 cm^−1^, 1235 cm^−1^ and 1735 cm^−1^ almost disappeared which prove that lignin, hemicellulose and C=O functional groups were largely removed from PV (Fig. 3). Fig. 3 indicates that the absolute-drying weight of PV (80 mm × 80 mm × 3 mm) has reduced from ∼4.5 g to ∼2.1 g after delignification and absolute-drying treatment.

**Fig. 4:**
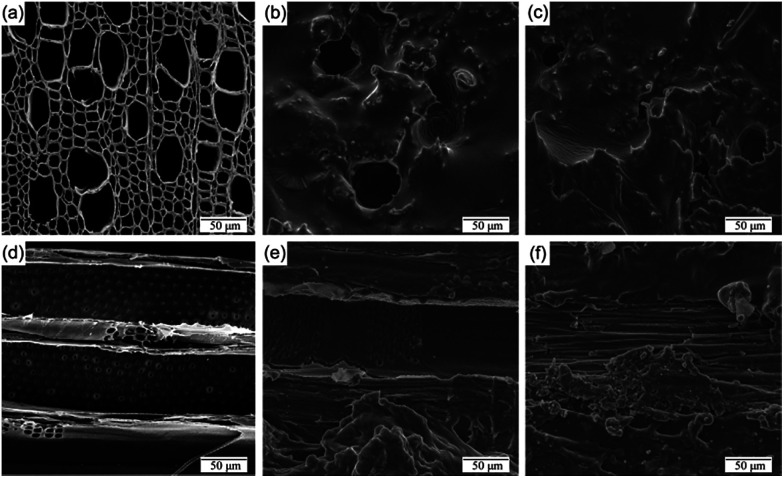
SEM image (a and d), reproduced with permission from China Pulp Paper (2017) and the Northwest Forestry University (2017), ref. 21 and 22, respectively, and (b, c, e and f) show the sample of PV before and after PU infiltration, respectively.

Transparent polymer infiltration is an important step in the production of TWC. Our TPC PU composite was examined using Quanta 450 scanning electron microscopy (FEI, US). Fig. 4 (a and d) show the scanning electron microscope (SEM) image of radial-cutting PV and longitudinal-cutting PV before PU infiltration. Fig. 4(b, c, e and f) show the SEM images of radial-cutting PV and longitudinal-cutting PV after PU infiltration. Comparing to the SEM images (a and d), SEM image (b, c, e and f) show that the microstructure is well-preserved after being filled with PU.

### 3.3 Flexibility and color-stability at high temperature

After the gaps between the cellulose fiber network had been infiltrated by PU and these polymers hardened, the resulting TPC PU (80 mm × 20 mm × 3 mm) had improved flexibility and color-stability against high temperatures compared to epoxy resin-infiltrated TPC (TPC ER, 80 mm × 20 mm × 3 mm).

**Fig. 5:**
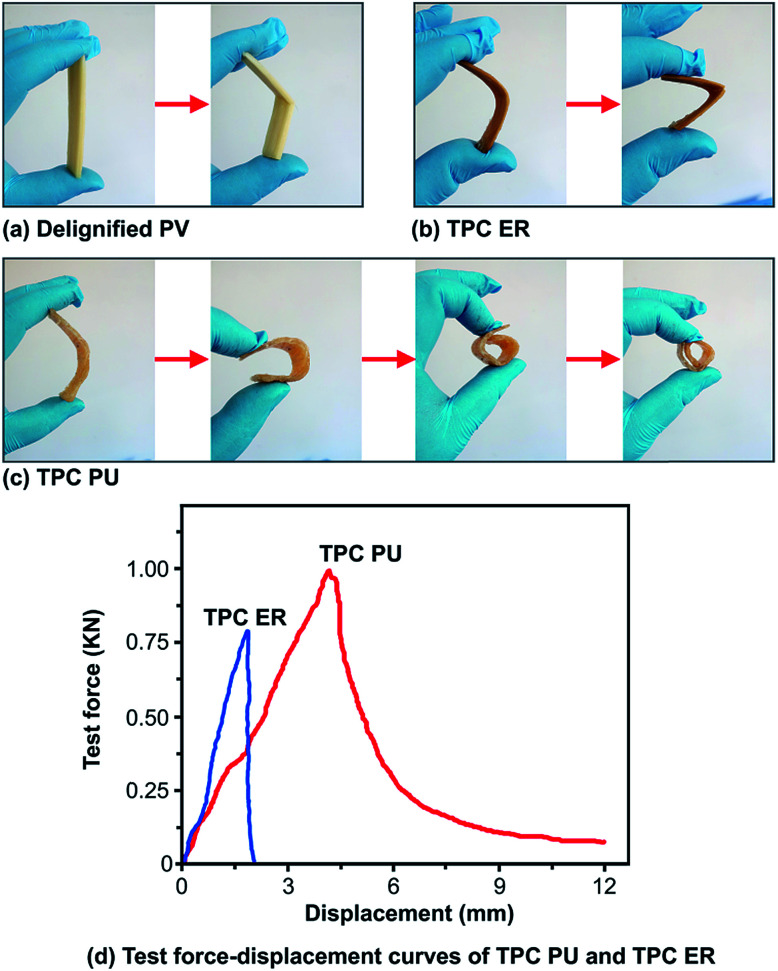
(a) After the sample of PV being treated by removing lignin and bleaching cellulose, it breaks upon bending. (b) After the sample of PV being treated by removing lignin, bleaching cellulose and infiltrating epoxy resin, it breaks upon bending. (c) After the sample of PV being treated by removing lignin, bleaching cellulose and infiltrating PU, it becomes more flexible upon bending. (d) Test force–displacement curves about tensile strength, the displacement scale of TPC PU is about 7.5 times than TPC ER.

Flexible wood has increasingly attracted scientific interest due to its wide applications.^23^ After removing lignin, bleaching the cellulose and infiltrating with PU, TPC PU has better flexibility including more elongation before breaking than TPC ER. Fig. 5(a) shows that the sample of PV breaks upon bending after removing lignin and bleaching with cellulose. Fig. 5(b) shows that the sample of PV breaks upon bending after removing lignin, bleaching cellulose and infiltrating with epoxy resin. Fig. 5(c) indicates that the sample of PV becomes more flexible upon bending after removing lignin, bleaching with cellulose and infiltrating with PU. Its bending radius is about 2.4 mm. The tensile strength of samples were tested by SmartTest (Joyrun, China). Fig. 5(d) shows the tensile strength test force–displacement curves. According to Fig. 5(d) and the equation for calculating elongation at break:Elongation at break (%) = *L*′ − *L*/*L* × 100%

*L* of samples are 80 mm. After test of tensile strength, *L*′ of TPC PU is about 92 mm and its elongation at break could be about 15%, *L*′ of TPC ER is about 82 mm and its elongation at break is about 2.5%.

**Fig. 6:**
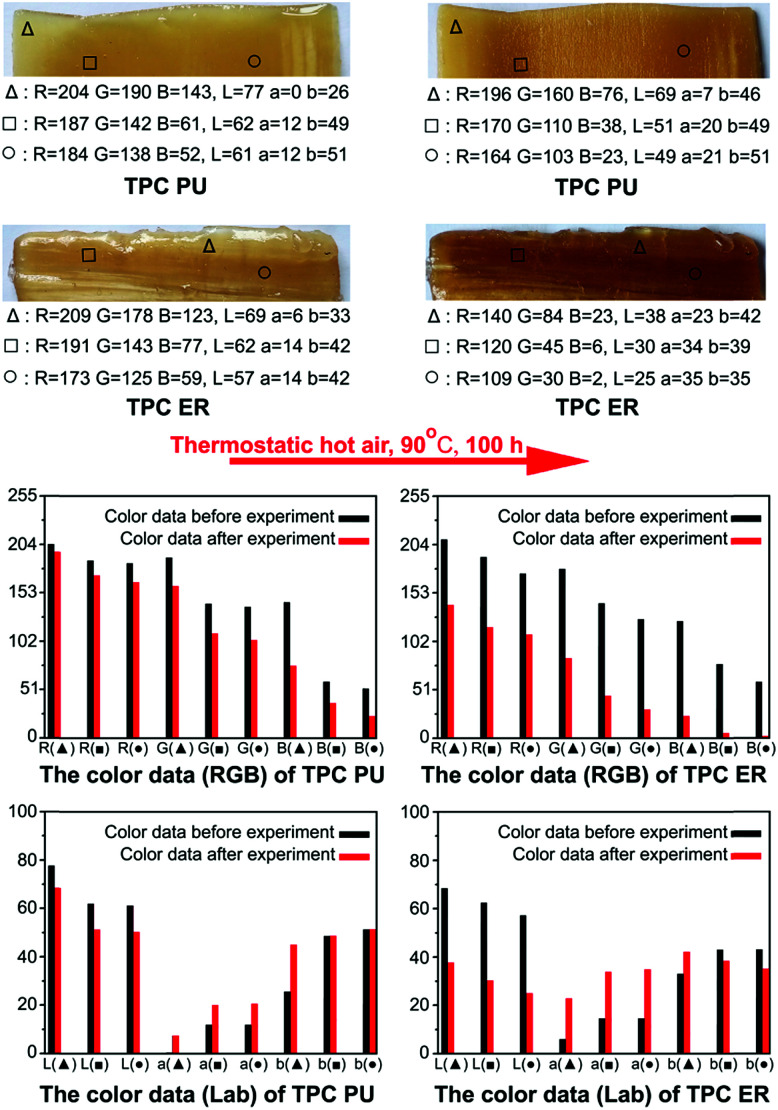
According to their respective change amplitude of RGB data and lab data before and after the hot air drying experiment, the color changes of TPC PU are less than TPC ER.

The transparency of TPC PU is more stable than TPC ER at high temperatures. We used Photoshop software (Adobe, US) to collect RGB data and Lab data of the color at three positions (▲, ■, ●) in photos of TPC PU and TPC ER before and after thermostatic hot air drying at 90 °C for 100 h. The hot air drying experiment involved a DGG-9203A electro-thermostatic blast oven (SLIC, Shanghai, China), and these photos of TPC PU and TPC ER were obtained by LiDE120 scanner (Canon, JP). As shown in Fig. 6, according to their respective change amplitude of RGB data and Lab data before and after the hot air drying experiment, TPC ER became darker.

### 3.4 Optical properties of TPC

TWC shows high optical transmittance and haze, with minor reflection on the outer surface.^1,24,25^ Research interest has increased in modifying wood cell and cell wall assemblies as well as cell wall components.^26^ Modified wood cells not only possess important engineering applications but also great potential in new technology fields.^27^ Surface modification of the wood cell wall will help to tune light scattering properties.^16^ The modified wood cell wall introduced strong scattering, resulting in diffused luminescence from embedded quantum dots.^28^

**Fig. 7:**
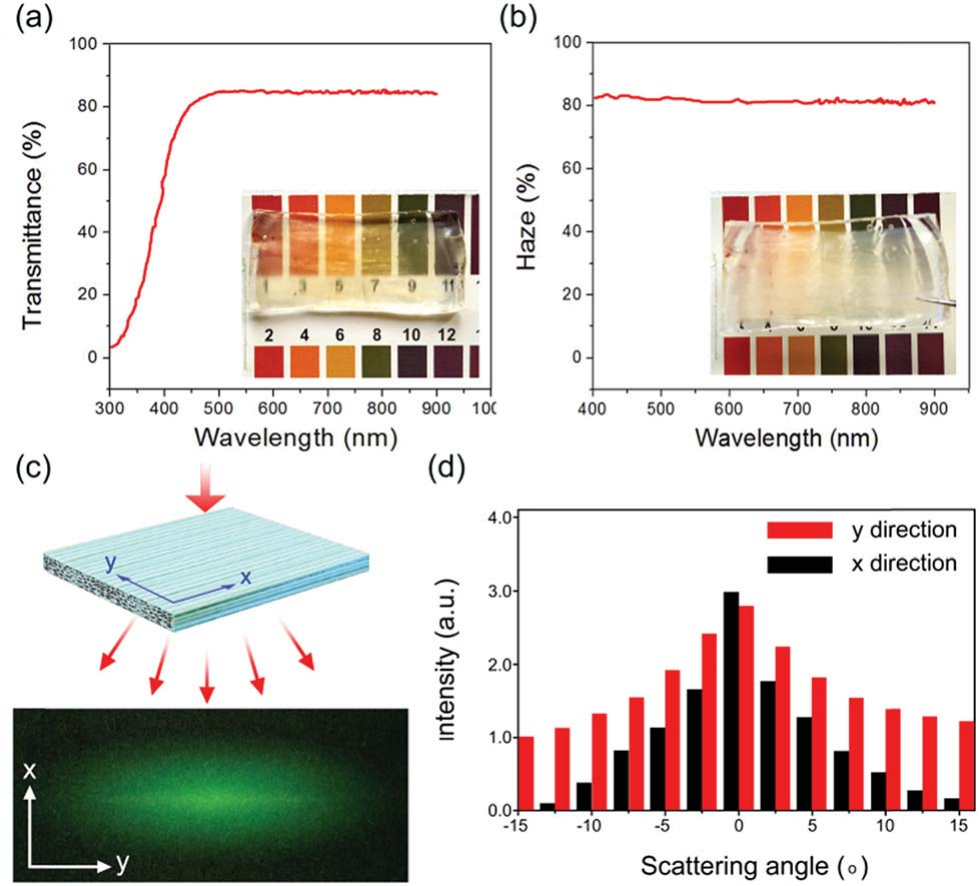
(a and b) After PU infiltration, the poplar–polymer composite with transmittance of 85%, haze of 83% was obtained. (c and d) Compared to the *x* direction, *y* direction has higher refractive index fluctuation.

In TPC, the light is diffracted in all directions by modifying its cell wall, and the optical haze is due to its structural anisotropy. The interface is critical for optical transmittance of TWC.^29,30^ The interfacial space of TPC could be increased in a reasonable range by removing lignin, improving the limited swelling of cellulose fiber network and infiltrating with PU. Between the cellulose fiber network of the poplar–polymer composite, the interfacial space linking lumen is also the light pathway for optical transmittance. Transmittance and haze were obtained by WGT-S transmittance and haze tester (SGIC, Shanghai, China). Fig. 7(a and b) show that the TPC with transmittance of 85%, haze of 83%. A photodiode power sensor S130C (Thorlabs, US) was used to record the scattered light intensity distribution in both the *x* and *y* directions. Fig. 7(c and d) indicate that the TPC with anisotropic light diffraction, and lower refractive index fluctuation in the *x* direction was obtained due to the aligned cellulose fibres in this direction.

TPC is a kind of eco-friendly TWC due to poplar being the most widely distributed and adaptable tree species in the world. The lignin of poplar was removed by the solution that included KOH and deionized water, and the black liquor could be converted into compound potassium fertilizer after being neutralized by H_3_PO_4_. After these gaps between its cellulose fiber network had been infiltrated, PU and these polymers have been hardened, this kind of TPC shows flexibility, color-stability against high temperature, and optical properties.

## 4. Conclusions

The PU-infiltrated TPC (TPC PU) was prepared by KOH delignification, NaClO bleach, C_2_H_6_O limited swelling, and PU infiltration. This kind of TPC PU is from an farmed poplar and the black liquor side-product could be turned into a fertilizer. TPC PU is better than TPC ER in terms of color-stability at higher temperatures, and flexibility – its elongation at break was about 15%. It also provided transmittance of 85%, haze of 83%, and anisotropic light diffraction. Furthermore, our future work will pay more attention to reduce the time cost in the preparation of TPC PU, to relieve the loss of poplar in the process of production, and to improve its volume and quality.

## Conflicts of interest

There are no conflicts to declare.

## Supplementary Material
